# Topical Formulation of Noscapine, a Benzylisoquinoline Alkaloid, Ameliorates Imiquimod-Induced Psoriasis-Like Skin Lesions

**DOI:** 10.1155/2022/3707647

**Published:** 2022-04-22

**Authors:** Fahimeh Nourbakhsh, Seyed Hadi Mousavi, Pouria Rahmanian-Devin, Vafa Baradaran Rahimi, Hassan Rakhshandeh, Vahid Reza Askari

**Affiliations:** ^1^Medical Toxicology Research Centre, Faculty of Medicine, Mashhad University of Medical Sciences, Mashhad, Iran; ^2^Pharmacological Research Center of Medicinal Plants, Mashhad University of Medical Sciences, Mashhad, Iran; ^3^Department of Cardiovascular Diseases, Faculty of Medicine, Mashhad University of Medical Sciences, Mashhad, Iran; ^4^Applied Biomedical Research Center, Mashhad University of Medical Sciences, Mashhad, Iran; ^5^Department of Pharmaceutical Sciences in Persian Medicine, School of Persian and Complementary Medicine, Mashhad University of Medical Sciences, Mashhad, Iran; ^6^Department of Persian Medicine, School of Persian and Complementary Medicine, Mashhad University of Medical Sciences, Mashhad, Iran

## Abstract

Psoriasis is considered an autoimmune inflammatory disease. The disease is spread and diagnosed by the infiltration of inflammatory mediators and cells into the epidermis. Recent theoretical developments have focused on the effectiveness of noscapine (NOS) as a potential alkaloid for being used as a valuable treatment for different diseases. In the present study, psoriasis-like dermatitis was induced on the right ear pinna surface of male *Balb/c* mice by topical application of imiquimod (IMQ) for ten consecutive days, which was treated with noscapine (0.3, 1, 3, and 10% w/v) or clobetasol (0.05% w/v) as a positive control. The levels of ear length, thickness, severity of skin inflammation, psoriatic itch, psoriasis area severity index (PASI) score, and body weight were measured daily. On the 10^th^ day of study, each ear was investigated for inflammation, fibrosis, proliferation, and apoptosis using histopathological (H&E and Masson's trichrome staining) and immunohistochemistry (Ki67 and p53 staining) assays. Furthermore, the levels of inflammatory biomarkers were characterized by an enzyme-linked immunosorbent assay (ELISA). The results confirmed IMQ-induced psoriasis for five consecutive days. In contrast, noscapine significantly reduced the ear length, thickness, severity of skin inflammation, psoriatic itch and body weight, tumor necrosis factor-*α* (TNF-*α*), transforming growth factor-*β* (TGF-*β*), interferon-gamma (IFN-*γ*), interleukin 6 (IL-6), IL-17, and IL-23p19 in a concentration-dependent manner (*P* < 0.001–0.05 for all cases). Overall, topical noscapine significantly ameliorated both the macroscopical and microscopical features of psoriasis. However, further clinical investigations are required to translate the effects to clinics.

## 1. Introduction

Inflammation is defined by a series of events beginning with an induction phase, progressing to a maximum of inflammation, and then ending with a resolution phase [1–3]. Acute inflammation allows for rapid and complete immune activation, which is necessary for successful innate immunity defense [1–4]. It starts with detecting exogenous and endogenous surface receptors caused by mechanically, chemically, or biologically induced tissue damage, followed by the recruitment of effector cells, which also orchestrates an inflammatory reaction defined by the release of lipid and protein-based mediators of inflammation [5].

Psoriasis is an autoimmune and chronic inflammatory disease in which keratinocytes are impressed with significant proliferation and abnormal differentiation [6]. The infiltration of inflammatory cells and related inflammatory mediators characterizes the disease into the epidermis and causes severe changes in the vascular system of the dermis [7]. Patients typically describe scaling of the body surface, itching, erythema, burning, and bleeding as symptoms. The prevalence of this disease globally is reported by 2-3% (more than 125 million people in the world), which is probably the most common autoimmune skin disease [8]. Both genetics and environmental factors are contributing to the disease [9]. In this regard, family history has been reported in approximately 30% of psoriatic patients. These patients have also observed polymorphisms of genes that regulate angiogenesis and inflammation [10, 11]. The entry of various immune cells and the production of inflammatory and pro-inflammatory cytokines in the epidermis intensify the inflammation in this area and develop psoriasis lesions [12]. Studies have shown that CD4^+^ and CD8^+^ T-cells play a fundamental role in the pathogenesis of psoriasis, initiating inflammation, and keeping inflammation in place.

On the other hand, the critical role of T-cells in psoriasis has been proven [13]. Although the exact pathogenesis of this disease is not yet fully understood, there is growing evidence that interleukin (IL)-23/IL-17A has a significant impact on the pathogenesis of the disease. In addition, several studies have demonstrated that the primary source of IL-17A production in the skin is *γδ*-T cells producing IL-17A by stimulating IL-23 [14]. Many inflammatory cytokines, including IL-23, IL-17A, IL-20, IL-22, IL1-*β*, IL-6, and TNF-ɑ (tumor necrosis factor-*α*), act as a complex in the disease development [14]. Imiquimod (IMQ), a ligand for toll-like receptors (TLRs)-7/8 and a potent activator of the immune system, is used to treat genital and anal warts (caused by human papillomaviruses, HPV) selected as a psoriasis experimental design [15, 16]. In this regard, experimental data have shown that IMQ causes human-like dermatitis similar to psoriasis in animal studies. It was demonstrated that IMQ used in the treatment of active warts and keratosis could activate dendritic cells, plasma dendritic cells (PDCs), natural killer (NK) cells, and T lymphocytes to produce inflammation by interferon (IFN)-*γ* and IL-17/IL-23 secretions [14]. The experimental validation of this model might pave the way for novel treatment approaches that target upstream processes in psoriasis etiology [17]. Due to the use of this drug on the skin of mice, it causes the rapid entry of PDCs into the site. In fact, this drug's antiviral and antitumor activity is mainly mediated by activation of TLR-7 and TLR-8 (expressed by monocytes, macrophages, and PDCs) [18]. Although low doses of methotrexate and other suggested treatments are used weekly for psoriasis, numerous adverse effects have been reported for these medications, including hematological and gastrointestinal disorders such as abdominal discomfort, stomach pain, nausea and vomiting, and drowsiness [19, 20]. Therefore, the use and findings of medicines that control the severity of psoriasis with the least amount of adverse effects are a priority in the treatment and alleviation of psoriasis.

Noscapine is a benzyl alkaloid isoquinoline found in dark poppy plants with no opioid-like adverse effects [21–23]. However, it has been reported to inhibit cell proliferation in a wide range of cancer cells, even in many drug-resistant species [23–25]. Furthermore, noscapine has been demonstrated to be safe and to have no toxic effects on humoral or cell-mediated immunity. Along with its antitussive and antistroke effects, these findings generated an interest in establishing other roles of noscapine. The pharmacology of noscapine, as well as its effects on cellular signaling pathways, the mitotic spindle, and centrosome clustering, as well as its use as an antimalarial and cough suppressant, and its exceptional potential as a treatment for polycystic ovarian syndrome, strokes, and a variety of malignancies, are all detailed [22, 23]. Moreover, it has been indicated that noscapine could reduce the levels of inflammatory cytokines, such as IL-2, TNF-*α*, and IL-6, in different *in vitro* and *in vivo* models [22–25]. Therefore, in this study, we tried to evaluate the effectiveness of topical administration of noscapine in the animal model of IMQ-induced psoriasis with several macroscopic (such as ear length, thickness, severity of skin inflammation, psoriatic itch, psoriasis area severity index (PASI) score, and body weight), histopathologic (Hematoxylin and eosin, H&E, for inflammation and inflammatory cells infiltration levels, and Masson's trichrome for the fibrosis level), immunohistochemistry (Ki-67 as a marker of cell proliferation and p53 as a marker of cell apoptosis), and molecular (cytokines production) methods.

## 2. Materials and Methods

### 2.1. Chemicals and Reagents

Noscapine powder (purity 99.5%) was of analytical grade and bought from FaranShimi® Company, Hamedan, Iran. Cold cream was of commercial grade and obtained from Farabi® Company, Tehran, Iran. Unless otherwise noted, all chemicals and reagents were acquired from Sigma-Aldrich (St. Louis, MO, USA). Furthermore, 5% w/w imiquimod propionate cream patches was prepared from Aldara, MEDA, Sweden. The cytokine chemical and reagent characterizations were acquired from the eBioscience® company (St. Louis, MO, USA). Enzyme-linked immunosorbent assay (ELISA) kits were obtained for TNF-*α*, TGF-*β*, IFN-*γ*, IL-10, IL-6, IL-17A, and IL-23p19. Immunohistochemical examination was performed using the antibodies against Ki-67 protein (ZYTOMED) and p53 protein (GenomeMe) based on the kit protocols.

### 2.2. Preparation of Noscapine Cream

To prepare noscapine cream in the lowest dose (0.3 %w/w), 300 mg of noscapine was levigated in 2 mL of glycerin and then mixed with cold cream to have the final mixture of 100 g. Similarly, further doses of 1%, 3%, and 10% w/w of noscapine cream were prepared with the same method and different levels of noscapine. The final cream had good stability and illustration and was in a smooth consistency.

### 2.3. Animal Husbandry and Ethical Statements

Forty-two male inbred *Balb/c* mice (weighing 25.2 ± 4.6 g) were obtained from the Faculty of Medicine Animal Laboratory, Mashhad University of Medical Sciences, Mashhad, Iran, and then randomly divided into six groups for this research (*n* = 7/group). Animals were housed in cages at 22 ± 1°C along with temperature conditions of 19–26°C, relative humidity of 70–70%, the light intensity of 300 lux, and exposure of 12 light/dark cycles. All animals had free access to food and tap water. Three or four mice were housed in each cage at better condition, which measured 206 × 365 × 140 mm. Moreover, the mice were acclimatized for one week before starting the study. In fact, all animal procedures were carried out in compliance with the National Institutes of Health's Guide for the Use and Care of Laboratory Animals guidelines of institutional guidelines. Moreover, the Mashhad University of Medical Sciences Ethical Committee approved all procedures involving animals based on the policies of animal experiments and care (Ethical approval code: 990587, Approval date: 2020–09–07, Approval ID: IR.MUMS.MEDICAL.REC.1399.463).

### 2.4. Psoriasis Induction and Experimental Protocol

To induce psoriasis from the first day, a certain amount of 5% w/w IMQ cream (10 mg/cm^2^ of skin) was used on the right ear pinna of mice in each group. Daily photographs were taken to record changes in skin lesions and were scored based on PASI scores, inflammation, thickness, and redness of mice's right ear pinna. Doses of medication and treatment were used for ten days. *Balb/c* mice were grouped as follows.

 
*Group I*: sham group, received a daily topical cold cream application as the vehicle on the right ear pinna.
 
*Group II*: negative control group, received a daily topical dosage of 5% IMQ cream (10 mg/cm^2^ of skin) on the right ear pinna (left ear pinna was untreated as the internal control), and after 30 min received a daily topical cold cream, as the vehicle, on the right ear pinna.
 
*Group III*: positive group, received a daily topical dosage of 5% IMQ cream on the right ear pinna (left ear pinna was untreated as the internal control), and after 30 min received a daily topical clobetasol cream (0.05 %w/w) on the right ear pinna.
 
*Group IV*: NOS 0.3% w/w, received a daily topical dosage of 5% IMQ cream on the right ear pinna (left ear pinna was untreated as the internal control), and after 30 min received a daily topical 0.3% w/w noscapine cream on the right ear pinna.
 
*Group V*: NOS 1% w/w, received a daily topical dosage of 5% IMQ cream on the right ear pinna (left ear pinna was untreated as the internal control), and after 30 min received a daily topical 1% w/w noscapine cream on the right ear pinna.
 
*Group VI*: NOS 3% w/w, received a daily topical dosage of 5% IMQ cream on the right ear pinna (left ear pinna was untreated as the internal control), and after 30 min received a daily topical 3% w/w noscapine cream on the right ear pinna.
 
*Group VII*: NOS 10% w/w, received a daily topical dosage of 5% IMQ cream on the right ear pinna (left ear pinna was untreated as internal control), and after 30 min received a daily topical 10% w/w noscapine cream on the right ear pinna.

All doses were calculated based on the preliminary study with a three-fold increment in dose. An illustration of the experimental procedures is shown in Table 1.

### 2.5. Scoring Severity of Skin Inflammation

The levels of erythema, thickness, and scaling on the afflicted ear pinna surface were used to calculate PASI (Psoriasis Area and Severity Index) scores. On a four-point scale, PASI was calculated for each mice (0 = none; 1 = slight; 2 = moderate; 3 = marked; 4 = extremely marked). The total score was measured by the combined scores (scaling, erythema, and thickness). Ear thickness and ear length were measured in both ears every day with digital calipers (BEC, China). These factors are the criteria for the amount of epidermal growth and inflammation was measured by the rise in ear thickness and inflammation [26].

### 2.6. Behavioural Tests of IMQ-Induced Psoriatic Itch

Mice were acclimated to a plexiglass recording arena twice for 60 minutes each time before being tested. In addition, mice were recorded from above for 60 minutes two hours following each topical administration. A scratch bout was characterized as one or faster back-and-forth hind paw motions aimed at and touching the treated region, culminating in toe licking or biting or planting of the hind paw on the floor. Grooming motions and posterior paw movement directed away from the treatment region (e.g., ear scratching) were not counted [27].

### 2.7. Evaluating the Mice's Spleen, Ear, and Body Weight

At the end of the study, animals were sacrificed, and the tissues were dissected and kept at −70°C. In this way, the spleen and ear of each mouse were isolated, weighed and then photographed, and stored in a freezer with −70°C or 10% v/v formalin for further examinations. In addition, the spleen and ear index were calculated by dividing the weight of the spleen or ear of each mouse by the bodyweight of the mouse on the last day.

### 2.8. Histopathological Examination

Right ear pinnas were selected as a tissue sample and then were fixed in 10% v/v neutral-buffered formalin. Samples were placed separately in 10% v/v formalin in a Falcon-tube and delivered to the pathology laboratory. The samples were immersed in paraffin blocks and finally cut to a thickness of 4 *μ*m. Hematoxylin and eosin (H&E, evaluating inflammation and inflammatory cells infiltration levels) and Masson's trichrome (evaluating fibrosis level) stainings were considered for each sample and photographed with a digital camera microscope [28–30]. The ear was considered for H&E and Masson's trichrome stainings.

Moreover, the spleen and cervical spinal cord (C3–C4) was also selected for H&E staining investigation, including inflammation and cellular infiltrations. The stained tissues were photographed with a digital camera microscope (Nikon, Eclipse E200). The pathologic changes in the ear pinna of various groups were included: acanthosis, parakeratosis, and thickening of the subepidermal layer. These factors were evaluated semi-quantitatively on a scale of 0–10 (0 = No clinical signs or change, 1 = Partial inflammation, 2 = Slight inflammation and tissue damage in mice, 3 = Increased levels of inflammation and tissue destruction of the right ear pinna, spinal cord and increased spleen leukocytes, 4 = Low increase in epidermis thickness and inflammation, 5 = Moderate inflammation, ear pinna thickness, and low parakeratosis 6 = Moderate inflammation, moderate ear pinna thickness, epidermal parakeratosis 7 = Increasing inflammation, ear pinna thickness, epidermal parakeratosis, and a low score of hyperplasia of the epidermis 8 = Ear pinna thickness and inflammation, marked areas of necrosis with vacuolization of cells, epidermal parakeratosis, 9 = Ear pinna thickness and inflammation, parakeratosis of the epidermal layer, 10 = Maximum inflammation, increased inflammatory infiltration and hyperplasia of the epidermis and acanthosis) [26, 31].

### 2.9. Immunohistochemistry Examination

Immunohistochemistry (IHC) [32] is the method for detection of a specific antigen in a tissue, cell, or particular phase of a cell life cycle based on antigen-antibody detection using light microscopy [33]. In the present study, we performed IHC for detecting the Ki-67 as a marker of cell proliferation and the p53 as a marker of cell apoptosis in the right ear pinnas based on their kits' manual [32, 33]. The basic steps of the IHC-P protocol are as follows: (1) Fixing and embedding the tissue, (2) cutting and mounting the section, (3) deparaffinizing and rehydrating the section, (4) antigen retrieval, (5) immunohistochemical staining, (6) counterstaining (if desired), (7) dehydrating and stabilizing with mounting medium, and (8) viewing the staining under the microscope. These factors were semiquantitatively scaled of 0–10 (0 = no change from untreated ears to 10 = maximum).

### 2.10. Total Protein Measurement Method

The Bradford protein assay was carried out to quantify the total protein concentration in each sample [2, 3, 34, 35]. In this regard, first, the Coomassie brilliant blue G-250 dye (10 mg) was dissolved in 50 ml ethanol 96%. Then, phosphoric acid 85% v/v (10 ml) was added, and the volume of the solution was increased to 100 ml. Thereafter, bovine serum albumin (4 mg/ml) solution was prepared as a standard curve. Then, after sample pouring (20 *μ*l), a Bradford reagent (200 *μ*l) was added to the 96-well microplate. Finally, the light absorption was read at 595 nm with a microplate reader after 5 minutes [2, 3, 28, 35].

### 2.11. Cytokine Assessments

The right ear pinnas of the mice were excised and kept at −70°C to evaluate cytokine measurement in skin tissue. The skins were homogenized at 4°C in tissue protein lysis buffer. In the following, the supernatants were selected for measuring the concentration of cytokines such as TNF-*α*, TGF-*β*, IFN-*γ*, IL-10, IL-6, IL-17A, and IL-23p19. ELISA was performed under the manufacturer's instructions based on the kits' protocols [2, 3, 28, 35].

### 2.12. Statistical Analysis

Data were analyzed using GraphPad Prism ® software version 8 (GraphPad Software, San Diego, CA) and presented based on the natures of parametric or non-parametric as means ± SD or median ± range (for the pathological score, including acanthosis, parakeratosis, thickening of subepidermal, histology score), respectively. The normality test was done for parametric data, based on the Kolmogorov–Smirnov and Bartlett's tests that assess the homogeneity of variances. Thereafter, comparisons between groups were carried out using a two-way analysis of variance (ANOVA) followed by a Sidak's multiple comparisons *post hoc* test. Repeated measures two-way ANOVA was done with the following Dunnett-T_3_ multiple comparisons *post hoc* test for clinical scores. Non-parametric data were analyzed using the Kruskal–Wallis and Dunn's post-hoc multiple comparisons tests. The Brown–Forsythe test was also used if the SD was not equal and the statistical test in the equality of group variances based on performing an analysis of variance. Noteworthy, when *P* values (*P*) ≤ 0.05, 0.01, and 0.001 were considered statistically significant [2, 3, 28, 35]. The data and statistical analysis complied with the recommendations on experimental design, analysis and data sharing, and presentation in preclinical pharmacology [36–38]. A summary of the statistical analysis is shown in Table 2.

## 3. Results

### 3.1. Effects of Noscapine on the Ear Thickness, Erythema, Scales, and Ear Length

**Figure 1:**
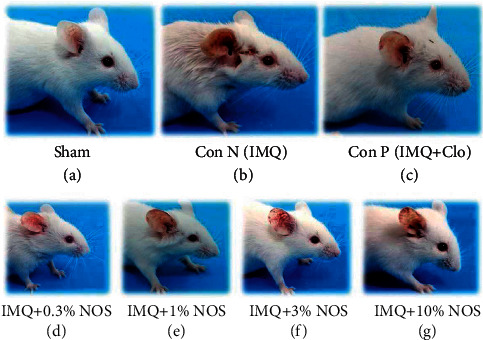
Severity of psoriasis-like symptoms in all groups for ten days was showing (a) sham group, (b) negative control group, (c) positive group, (d) sample group received IMQ plus 0.3% w/w NOS, (e) sample group received IMQ plus 1% w/w NOS, (f) sample group received IMQ plus 3% w/w NOS, and (g) sample group received IMQ plus 10% w/w NOS.

**Figure 2:**
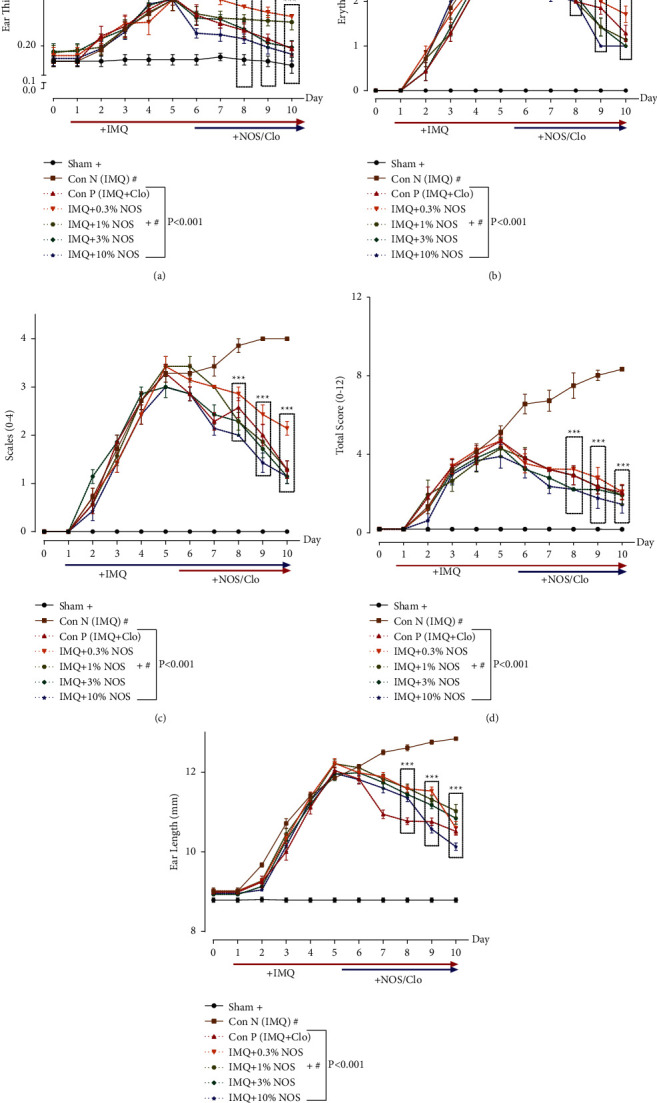
Phenotypical observations of ear pinna in treated mice groups. The symptoms of the thickness (a), erythema (b), scaling (c), the total score (d), and ear length (e) after commencing IMQ administration right ear pinna of the mice. The results are expressed as the mean ± SD (*n* = 7 in each group). The results indicate significant changes in the sham and negative control group (^*∗∗∗*^*P* < 0.001).

The right ear pinna of the mice showed symptoms of erythema, scaling, and thickening a couple of days after commencing IMQ administration (Figure 1). In the IMQ group (II), the severity of psoriasis-like symptoms steadily increased until day ten compared to the sham group (Figures 1 and 2).

Our results showed that the IMQ 5% application significantly increased the ear thickness compared to the sham group (*P* < 0.001, Figure 2(a)). In contrast, all concentrations of NOS (0.3, 1, 3, and 10% w/w) significantly and concentration-dependently reduced the level of the ear thickness compared to the IMQ-treated alone group (*P* < 0.001 for all cases). Furthermore, the trend of thickness reduction in the treated groups was observed from day seven at 0.3, 1, and 3% w/w of noscapine concentrations (*P* < 0.001 for all cases, Figure 2(a)) and from day six in NOS 10% w/w (*P* < 0.001, Figure 2(a)).

The results also showed an upward trend in inflammation and redness in all groups, up to day five of psoriasis induction. This study shows that all concentrations of NOS had a reducing effect on the amount of erythema compared to the IMQ-treated alone group (*P* < 0.001 for all cases, Figure 2(b)).

Scales (0–4) indicated the focal formation of inflamed and prominent plaques in different groups due to the overgrowth of skin epithelial cells. Scoring ranges are from zero to four based on each mouse's erythema, scaling, and thickening. Our results indicated that the application of different concentrations of NOS (0.3, 1, 3, and 10% w/w) significantly reduced the scale range from day eight compared to the control group (*P* < 0.001 for all cases, Figure 2(c)). However, NOS 3% w/w (2.429 ± 0.202, *P* < 0.001, Figure 2(c)) and NOS 10% w/w (2.143 ± 0.143, *P* < 0.001, Figure 2(c)) had a significant reduction effect on scales on the treatment groups from day seven (Figure 2(c)).

In addition, our findings also demonstrated that the total score in the control group received IMQ was significantly increased compared to the sham group (*P* < 0.001, Figure 2(d)). On the contrary, the application of different concentrations of NOS (0.3, 1, 3, and 10% w/w) and also Clo significantly diminished the total score from day eight compared to the control group (*P* < 0.001 for all cases, Figure 2(d)).

The results showed a significant increase in ear length during the use of IMQ compared to the sham group (*P* < 0.001, Figure 2(e)). In contrast, the application of different concentrations of NOS (0.3, 1, 3, and 10% w/w) significantly attenuated the ear length from day eight compared to the control group (*P* < 0.001 for all cases, Figure 2(e)).

### 3.2. Effect of Noscapine on the Spleen and Body Weight

**Figure 3:**
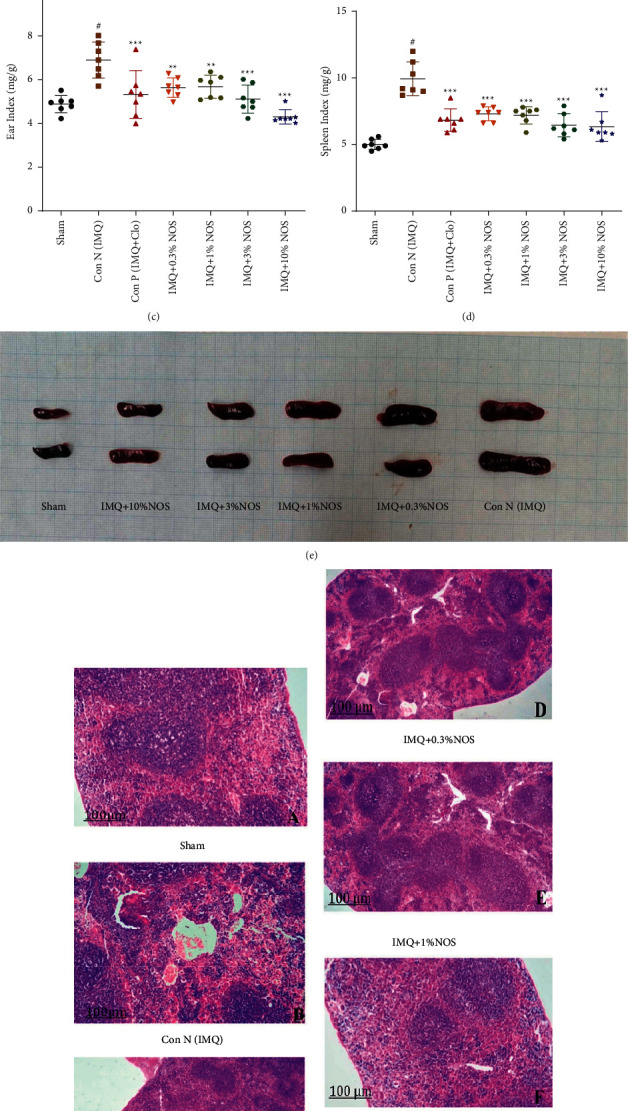
Investigation of mice in terms of body weight (a), the weight of the spleen (b), ear index (dividing weight of the ear to the bodyweight) (c), the spleen index (dividing weight of the spleen to the bodyweight) (d), representative photos of the spleen (e), and pathological investigation of the spleen (f), respectively showed in Figures 3(a)–3(f). The results are expressed as the mean ± SD (*n* = 7 in each group). The results indicate significant changes compared to the negative control group (^*∗∗∗*^*P* < 0.001, ^*∗∗*^*P* < 0.01 and ^*∗*^*P* < 0.05).

The results showed that IMQ 5% w/w on the ear led to a significant increase in the body weight compared to the sham group (*P* < 0.001, Figure 3(a)). However, different concentrations of NOS (1, 3, and 10% w/w) meaningfully and concentration-dependently reduced the elevated body weight compared to the control group (*P* < 0.001 to 0.05 for all cases, Figure 3(a)).

The application of IMQ 5% w/w on the ear resulted in a significant elevation in the spleen weight compared to the sham group (*P* < 0.001, Figure 3(b)). The use of each Clo (*P* < 0.05), NOS 1% w/w (*P* < 0.01), NOS 1% w/w (*P* < 0.001), NOS 3% w/w (*P* < 0.001), or NOS 10% w/w (*P* < 0.001) notably reduced the elevated spleen weight compared to the control group (Figure 3(b)).

Ear (Figure 3(c)) and spleen (Figure 3(d)) indices were calculated by dividing the net weight of each respected organ by the body weight. As a result, both ear (*P* < 0.001, Figure 3(c)) and spleen (*P* < 0.001, Figure 3(d)) indices were increased in the IMQ control group compared to the sham group. Experimentally, using each Clo or NOS (0.3, 1, 3, and 10% w/w) notably reduced the elevated ear (*P* < 0.001–0.01 for all cases, Figure 3(c)) and spleen (*P* < 0.001 for all cases, Figure 3(d)) indices compared to the control group.

Additionally, Figure 3(e) represents the spleen length of each group, including A: negative control group, B: positive group, C: sample group received IMQ plus 0.3% w/w NOS, D: sample group received IMQ plus 1% w/w NOS, E: sample group received IMQ plus 3% w/w NOS, F: sample group received IMQ plus 10% w/w NOS. Moreover, Figure 3(f) shows the spleen's normal white pulp and abnormal red pulp in various groups.

### 3.3. Effect of Noscapine on the Scratching Behavior

**Figure 4:**
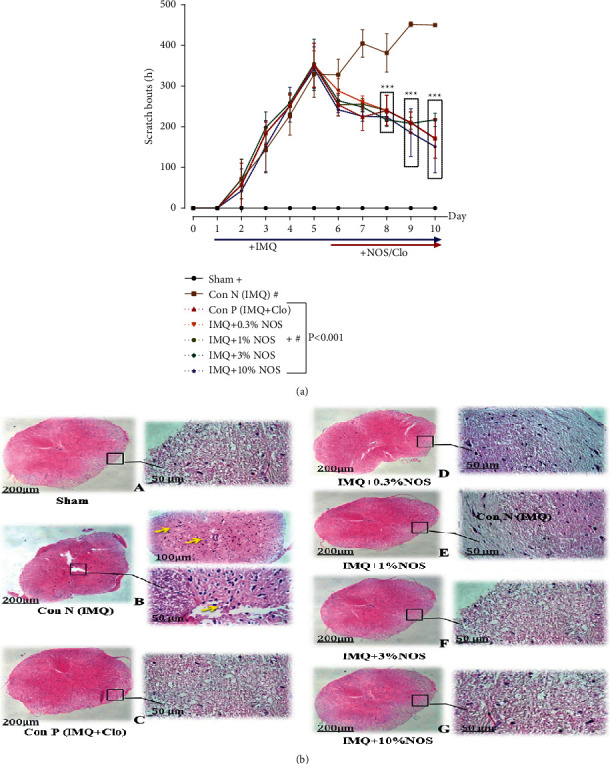
Number of scratching behavior in IMQ-induced psoriasis-like skin (a) and also H&E staining of cord sections revealed marked areas of necrosis with vacuolization of cells (b). The results are expressed as the mean ± SD (*n* = 7 in each group). The results indicate significant changes compared to the negative control group (^*∗∗∗*^*P* < 0.001).

The spontaneous scratch bouts were continuously greater in IMQ-treated mice than in control animals and steadily increased with time compared to the sham group (*P* < 0.001, Figure 4(a)). The rate of pruritus in the groups receiving IMQ increased sharply, while this rate increased significantly on days two to five and remained at its maximum until day five of psoriasis induction (Figure 4(a)). Reduction of pruritus was observed considerably in treated mice from day seven at doses of 1% w/w (255.7 ± 5.2), 3% w/w (248.5 ± 4.5), and 10% w/w NOS (196.2 ± 29.2) (*P* < 0.001 for all cases, Figure 4(a)). H&E staining of spinal cord sections revealed marked areas of necrosis with vacuolization of cells (Figure 4(b)).

### 3.4. Effect of Noscapine on the Concentration of the Cytokines

**Figure 5:**
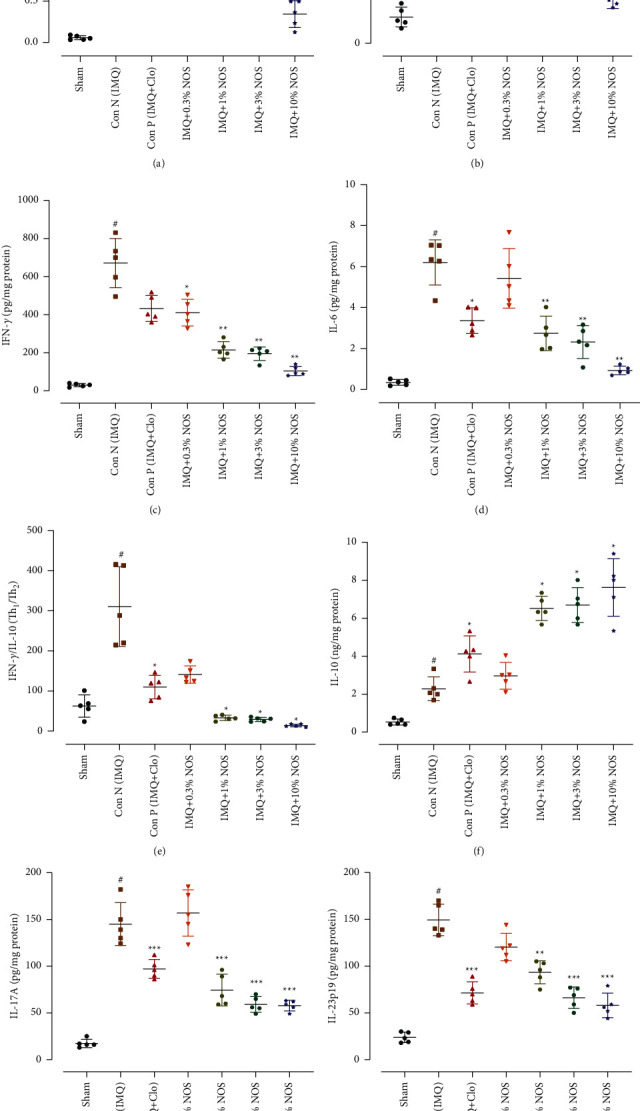
Concentration of cytokines levels such as TNF-*α* (a), TGF-*β* (b), IFN-*γ* (c), IL-6 (d), IFN-*γ*/IL-10 (e), IL-10 (f), IL-17A (g), and IL-23p19 (h), respectively, showed in Figure 5(a)–5(h). The results are expressed as the mean ± SD (*n* = 7 in each group). The results indicate significant changes compared to the negative control group (^*∗∗∗*^*P* < 0.001, ^*∗∗*^*P* < 0.01, and ^*∗*^*P* < 0.05).

ELISA assays were used to assess inflammatory cytokines, essentials for inflammation in ear tissues of normal, IMQ-received, and NOS treated groups. In-ear skin samples from IMQ-received mice, levels of TNF-*α*-mediated cytokines, notably TGF-*β*, IL-6, IFN-*γ*, IL-17, and IL-23p19, were substantially increased compared to the sham group (*P* < 0.001 for all cases, Figures 5(a)–5(h)). In contrast, different concentrations of NOS (1, 3, and 10% w/w) significantly and concentration-dependently decreased in TNF-*α*, TGF-*β*, IFN-*γ*, IL-6, IFN-*γ*/IL-10, IL-17A, and IL-23p19 and also increased in the IL-10 expression in comparison to the control group (*P* < 0.001–0.05 for all cases, Figures 5(a)–5(h)).

### 3.5. Effect of Noscapine on the Histopathological Examination

**Figure 6:**
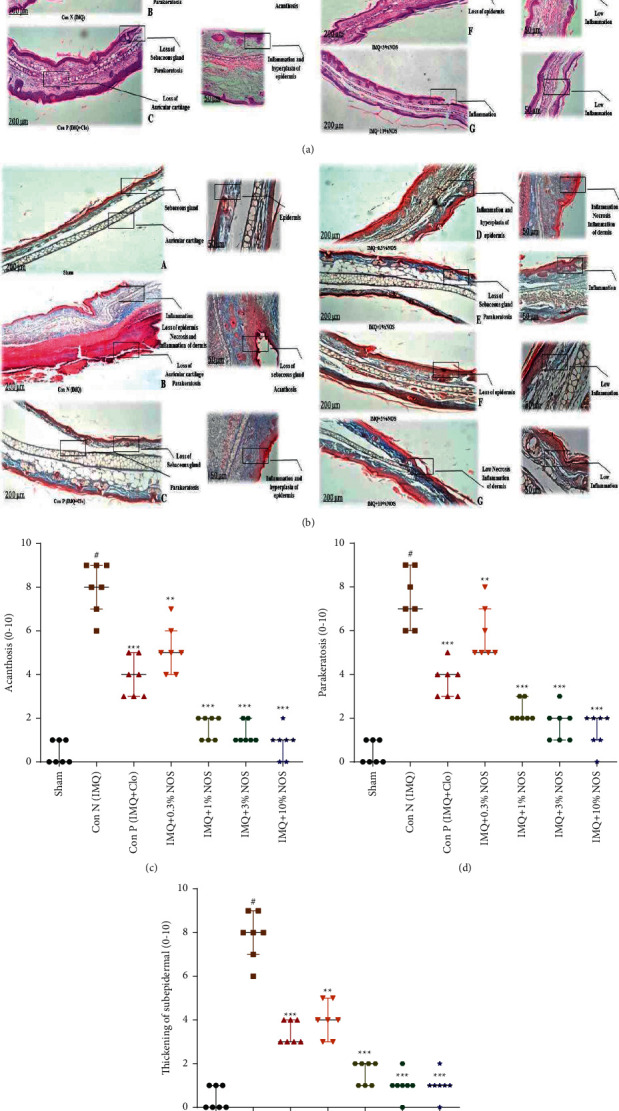
Hematoxylin and eosin (a) and trichrome staining (b) were presented for each sample. The pathologic changes in the ear pinna of various groups were included: acanthosis (c) parakeratosis (d) and thickening of the subepidermal layer (e) The data are expressed as the median ± IQR, (*n* = 7 in each group). The results also indicate significant changes compared to the negative control group (^*∗∗∗*^*P* < 0.001, ^*∗∗*^*P* < 0.01 and ^*∗*^*P* < 0.05).

H&E and Masson's trichrome staining (Figures 6(a) and 6(b)) of tissues were stained for each sample and photographed with a digital camera microscope (Nikon, Eclipse E200). Stained slices of IMQ-treated right ear pinnas were determined to match phenotypic findings and PASI score values. The pathologic changes in the ear pinna of various groups were acanthosis (Figure 6(c)), parakeratosis (Figure 6(d)) and thickening of the subepidermal layer (Figure 6(e)). These factors were evaluated semiquantitatively on a scale of 0–10 (0 = no change from untreated ears; 10 = maximum).

The IMQ-treated mice's right ear pinna sections revealed substantially enhanced acanthosis (*P* < 0.001, Figure 6(c)), parakeratosis, (*P* < 0.001, Figure 6(d)) and thickening of the subepidermal layer (*P* < 0.001, Figure 6(e)) in comparison to the control group (Figures 6(a) and 6(b)). The results also demonstrated that applying NOS (0.3, 1, 3, and 10% w/w), or Clo significantly reduced acanthosis (*P* < 0.001–0.01 for all cases, Figure 6(c)), parakeratosis (*P* < 0.001–0.01 for all cases, Figure 6(d)), and thickening of the subepidermal layer (*P* < 0.001 for all cases, Figure 6(e)) in comparison to the control group.

### 3.6. Effect of Noscapine on the Immunohistochemical Examination

**Figure 7:**
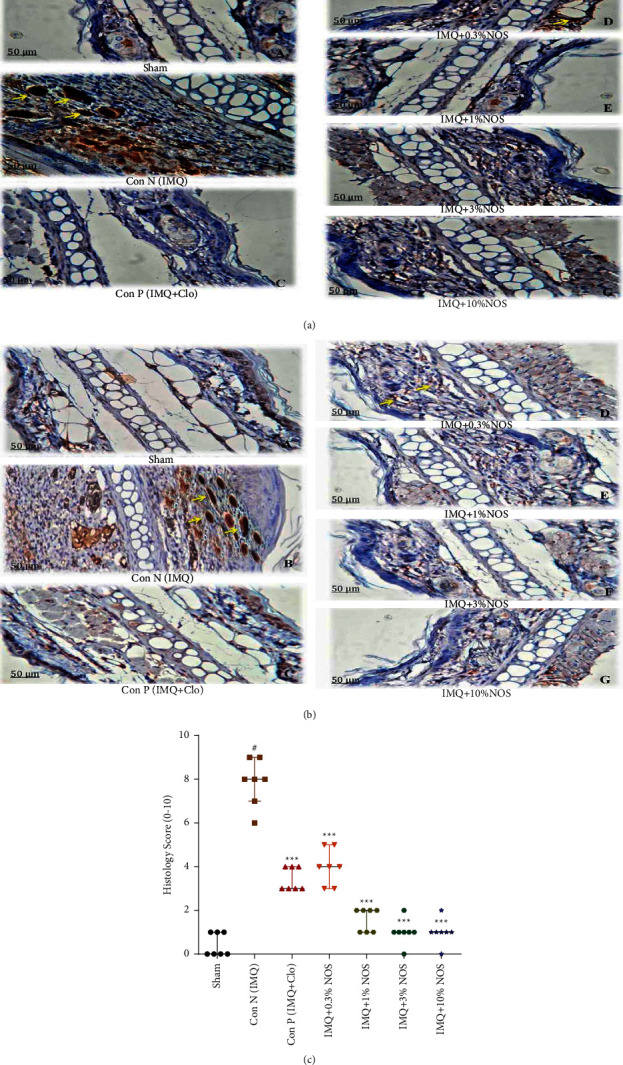
Immunohistochemistry (IHC) staining for p53 protein (a) and Ki-67 protein (b) as specific antigen in an ear tissue based on antigen-antibody reaction by light microscopy. The histopathological score of examined tissues (c) is expressed as the median ± IQR, (*n* = 7 in each group). The results also indicate significant changes compared to the negative control group (^*∗∗∗*^*P* < 0.001, ^*∗∗*^*P* < 0.01 and ^*∗*^*P* < 0.05).

Immunohistochemistry (IHC) staining was performed to detect p53 as a marker of cell apoptosis (Figure 7(a)) and Ki-67 as a cell proliferation marker (Figure 7(b)) based on the kit protocols by light microscopy. Positive p53 was presented in the subepidermal layer viewed by the brown nuclei of epidermal cells. Furthermore, positive Ki-67 was shown in the epidermis and considered by the brown nuclei of epidermal cells. Additionally, Figure 7(c) also presented a histopathological score of examined tissues semiquantitatively on a scale of 0–10 (0 = no change from untreated ears; 10 = maximum change in treated ears). In this regard, we found that the histology score was significantly increased in the IMQ-control group compared to the sham group (*P* < 0.001, Figure 7(c)). In contrast, applying NOS (0.3, 1, 3, and 10% w/w) or Clo significantly reduced the histology score compared to the control group (*P* < 0.001 for all cases, Figure 7(c)).

## 4. Discussion

To the best of our knowledge, this is the first study that evaluated the protective effects of noscapine against the IMQ-induced mouse model. As a result, we revealed that noscapine concentration-dependently and significantly reduced the inflammation and psoriatic manifestation of IMQ by lowering the ear thickness and length and modulating the apoptosis (p53) and cell proliferative (Ki-67) factors.

Since the prevalence of pruritus in patients with psoriasis has been reported to be 60%–90%, and many patients with psoriasis consider pruritus to be the most annoying symptom [39, 40], this study also examined and showed significant results of the therapeutic effects of noscapine in the pruritus induction model by IMQ. In line with the present investigation, numerous studies have evaluated the prevalence of pruritus in patients with psoriasis. Still, in general, only a focused treatment with a molecular signaling survey can relieve the itching associated with psoriasis [41]. Psoriasis is a chronic inflammatory and autoimmune disease in which epithelial cell proliferation increases compared to normal skin [42]. The effects of methotrexate and clobetasol on cell proliferation [43] and the impact of these drugs on the immune system justify the effectiveness of these drugs in this disease that some patients might be sensitive to during long-term treatment [44]. Noscapine as an old drug has significant clinical effects, including antitussive properties and cell proliferation. Hence, we tried to evaluate the effectiveness of topical administration noscapine in the animal model of psoriasis. Following the results of this research, it is possible to continue studies in the human phase [45].

The results showed that the reduction in thickness and skin inflammation occurred from day seven in all concentrations of noscapine. In fact, we found that noscapine can reduce fibrosis and also inflammation in the ear by reducing the inflammatory mediators (TNF-*α*, TGF-*β*, IFN-*γ*, IL-6, IL-17, and IL-23p19), increasing IL-10 as an anti-inflammatory cytokine and modulating the apoptosis (p53) and cell proliferative (Ki-67) factors. The present study's findings were consistent with the results of other studies that indicated that topical administration of capsaicin significantly inhibited skin inflammation [46]. This reduction in thickness was not significant in the positive control group receiving the clobetasol from day six of treatment, indicating the superiority of the topical form of noscapine over the clobetasol application. Similar results were obtained in the therapeutic effects of curcumin from day 6 of treatment in reducing inflammation and skin thickness in psoriasis-induced mice [47].

Numerous studies have reported the potent and significant antioxidant activity of noscapine [23, 48] in reducing the NF-*κ*B expression, which leads to a decrease in cytokine production by T-cells [23, 49, 50]. Currently, we demonstrated that noscapine considerably inhibited the pro-inflammatory mediator productions such as TNF-*α* or IFN*γ*. Our evidence suggests that noscapine has high antioxidant activity and substantially decrease the transcription of the TNF-*α* signaling indicator, resulting in a reduction in T-cell cytokine production. It was shown that TNF-*α*-mediated cytokines, TGF-*β*, IL-6, and IFN*γ* were considerably reduced in the noscapine treated groups than the positive control group. Our results align with previous studies that pointed out the anti-inflammatory properties of noscapine, primarily via modulating the NF-*κ*B signaling pathway in chemotherapeutic agents [23, 51]. Accordingly, noscapine treatment could impress the release of pro-inflammatory cells cytokines due to environmental stress and stress-activated protein kinases [52]. In this regard, similar researches show that TLR-mediated TNF-*α* and nitric oxide (NO) production in human and murine macrophages was inhibited by brominated noscapine analogues (Br-nos and Red-Br-nos) with no indication of cellular damage [23]. Brominated noscapine analogs also reduced cytokine/chemokine (a non-TLR ligand)-induced sterile inflammation [23, 53].

It has been reported that IL-10 production has therapeutic benefits as common anti-psoriatic treatments on several cell types [54]. In this context, the present research results showed that treatment with all concentrations of noscapine except 0.3% NOS increases the level of IL-10. Furthermore, the current study results showed the effectiveness of a high concentration of noscapine (10%w/w) in regulating the expression of pro-inflammatory cytokines. Although definite mechanisms and signaling pathways in reducing these cytokines are indeterminate yet [23, 54], we believe that noscapine's antiproliferative and anti-inflammatory effects act through the suppression of TNF-*α*/IFN*γ*, which results in more severe inflammation and keratinocyte proliferation. These findings are inconsistent with previous research that the anti-psoriatic effects of the Wannachawee recipe significantly inhibited the TNF-*α* and IFN*γ*-stimulated IL-17A, IL-22, and IL-23 secretion in HaCaT cells [26]. Although specific anti-inflammatory signaling pathways of noscapine in psoriasis induction are undefined, based on the current study results, we proposed that noscapine's antiproliferative and anti-inflammatory effect operates through the suppression of TNF-*α*/IFN*γ*/IL-23.

**Figure 8:**
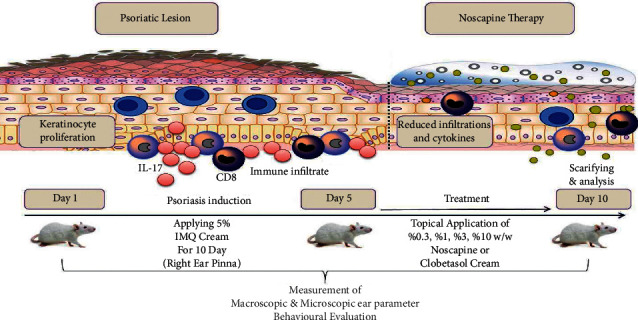
NOS alleviates IMQ-induced psoriasis-like skin lesions.

In conclusion, despite this investigation, it is well documented that noscapine can be considered as one of the therapeutic drugs in psoriasis disease and is widely evaluated in clinical trials studies.  Figure 8 shows a graphical abstract of the procedure and possible protective mechanism of NOS against IMQ-induced psoriasis-like skin lesions.

## Figures and Tables

**Table 1:** Illustration of the experimental procedures.

Protocol	Groups		Treatment day
(*n* = 7/each group) Psoriatic lesion and Noscapine therapy	Sham	Daily topical cold cream (10 mg/cm^2^ of skin)	Day 1–10
	Control negative	Daily topical dosage of 5% IMQ cream (10 mg/cm^2^ of skin)	
	Control positive	Daily topical dosage of 5% IMQ + Daily topical clobetasol cream (0.05 %w/w)	
	Treatment	Daily topical dosage of 5% IMQ + daily topical 0.3% w/w noscapine cream	
		Daily topical dosage of 5% IMQ + daily topical 1% w/w noscapine cream	
		Daily topical dosage of 5% IMQ + daily topical 3% w/w noscapine cream	
		Daily topical dosage of 5% IMQ + daily topical 10% w/w noscapine cream	

**Table 2:** Summarized protocols of the present study.

ANOVAs table	Time *F* (DFn, DFd)	Number of columns (intervention)	Number of rows (time)	Time × intervention	ANOVAs test
Thickness	*F* (10, 60) = 147.7 *P* < 0.0001, *N* = 11	*F* (6, 42) = 471.7	307.4	*F* (60, 420) = 70.32	Two way ANOVA Dunnet
Erythema	*F* (10, 60) = 352.2 *P* < 0.0001, *N* = 11	*F* (6, 36) = 177.4	11	*F* (60, 360) = 25.42	Two way ANOVA Sidak
Scales	*F* (10, 60) = 409.3 *P* < 0.0001, *N* = 11	*F* (6, 36) = 124.4	11	*F* (60, 360) = 19.18	Two way ANOVA Sidak
Total score	*F* (10, 60) = 922.8 *P* < 0.0001, *N* = 11	*F* (6, 36) = 827.4	11	*F* (60, 360) = 72.62	Two way ANOVA Sidak
Ear length	*F* (10, 60) = 467.5 *P* < 0.0001, *N* = 11	*F* (6, 36) = 363.5	11	*F* (60, 360) = 53.04	Two way ANOVA Dunnet
Scratch bouts	*F* (10, 420) = 509.3 *P* < 0.0001, *N* = 11	*F* (6, 36) = 230.3	11	*F* (60, 360) = 31.27	Two way ANOVA Dunnet
Body weight	*F* (2.970, 17.82) = 15.12 *P* < 0.0001	7	7	F (6, 36) = 3.490	One-way ANOVA Dunnet
Spleen weight	*F* (6, 42) = 16.19 *P* < 0.0001	1	7	—	One-way ANOVA Dunnet
Ear index	*F* (6, 42) = 10.62 *P* < 0.0001	1	7	—	One-way ANOVA Dunnet
Spleen index	*F* (6, 42) = 21.30 *P* < 0.0001	1	7	—	One-way ANOVA Dunnet
TNF-ơ	*F* (6, 18) = 53.55 *P* < 0.0001	7	35	—	Brown Forsythe test Dunnet T3
IL-10	*F* (6, 15) = 45.19 *P* < 0.0001	7	35	—	Brown Forsythe test Dunnet T3
TGF-*β*	*F* (6, 22) = 49.82 *P* < 0.0001	7	35	—	Brown Forsythe test Dunnet T3
IL-6	*F* (6, 15) = 31.8 *P* < 0.0001	7	35	—	Brown Forsythe test Dunnet T3
INF-ɣ	*F* (6, 10) = 57.52 *P* < 0.0001	7	35	—	Brown Forsythe test Dunnet T3
INF-ɣ/IL-10	*F* (6, 5) = 31.18 *P* < 0.0001	7	35	—	Brown Forsythe test Dunnet T3
IL-17A	*F* (6, 14) = 52.85 *P* < 0.0001	7	35	—	Brown Forsythe test Dunnet T3
IL-23p19	*F* (6, 23) = 54.29 *P* < 0.0001	7	35	—	Brown Forsythe test Dunnet T3

## Data Availability

The data used to support the findings of this study are available from the first author (F.N) upon reasonable request.

## Abbreviations

H and E: Hematoxylin and eosin
IFN-*γ*: Interferon-*γ*
IL-10: Interleukin 10
IL-17: Interleukin 17
IL-23p19: Interleukin 23p19
IL-6: Interleukin 6
IMQ: Imiquimod
NOS: Noscapine
PASI: Psoriasis area and severity index
PDCs: Plasmacytoid dendritic cell
TGF-*β*: Transforming growth factor-*β*
TLR: Toll-like receptor
TNF-*α*: Tumor necrosis factor-*α*.
